# Influenza B-Associated Acute Necrotizing Encephalopathy in a Previously Healthy Child: A Case Report of Fulminant Neurological Deterioration

**DOI:** 10.7759/cureus.110133

**Published:** 2026-06-02

**Authors:** Zainab Mahfoodh, Fatema Marzooq

**Affiliations:** 1 Radiology, Salmaniya Medical Complex, Manama, BHR

**Keywords:** acute encephalopathy, acute necrotizing encephalopathy, cytokine storm, immunomodulatory therapy, influenza b, intensive care, mri brain, pediatric encephalopathy, seizures, thalamic lesions

## Abstract

Acute necrotizing encephalopathy (ANE) is a rare, rapidly progressive, immune-mediated encephalopathy most commonly triggered by viral infections such as influenza, characterized by acute encephalopathy, seizures, and a high risk of severe neurological sequelae or death. We report a six-year-old previously healthy female who presented with several days of fever, reduced oral intake, progressive altered mental status, and new-onset seizures, requiring intensive care admission and mechanical ventilation due to worsening encephalopathy and respiratory compromise. Laboratory evaluation demonstrated systemic inflammation, and respiratory viral testing confirmed Influenza B infection. Neuroimaging revealed characteristic bilateral, symmetric lesions involving the thalami, external capsules, brainstem, and periventricular white matter with diffusion restriction, consistent with ANE. After exclusion of alternative infectious, metabolic, toxic, and autoimmune etiologies, a diagnosis of Influenza B-associated ANE was established. The patient was managed with high-dose corticosteroids, intravenous immunoglobulin, empirical antimicrobials, and comprehensive pediatric intensive care support, including antiseizure therapy and mechanical ventilation. Her course was complicated by ventilator-associated pneumonia, transient arrhythmias, and electrolyte disturbances, but she gradually improved with multidisciplinary management, achieving successful extubation and partial neurological recovery. Follow-up imaging demonstrated regression of acute lesions with residual changes. This case highlights the importance of early recognition, prompt neuroimaging, and aggressive immunomodulatory and supportive therapy in ANE, while underscoring its potential for significant morbidity despite intensive treatment.

## Introduction

Acute necrotizing encephalopathy (ANE) is a rare central nervous system (CNS) complication secondary to influenza or other viral infections, characterized by acute altered mental status, seizures, and rapid neurological deterioration, often progressing to severe disability or death. It is generally considered a parainfectious, immune-mediated disorder rather than a direct viral encephalitis, with pathogenesis thought to involve cytokine storm-mediated blood-brain barrier disruption. Initially described in pediatric patients of Asian descent, ANE is now recognized worldwide across diverse populations. Although most commonly reported in children, sporadic cases in adults have also been described, underscoring its broader clinical spectrum [1]. Systemic involvement may accompany the neurological syndrome, including laboratory abnormalities such as liver dysfunction, uremia, and hypoproteinemia. Cerebrospinal fluid analysis may show elevated protein, while findings are otherwise non-specific [2].

Neuroimaging is central to diagnosis, with the hallmark being multifocal, symmetric lesions involving the bilateral thalami, brainstem tegmentum, cerebellar medulla, and cerebral periventricular white matter, detectable on CT and more sensitively on MRI. Both gray and white matter can be involved, reflecting widespread disruption of the blood-brain barrier and associated cytotoxic and vasogenic edema [3]. These imaging features, in the appropriate clinical context of a preceding viral illness, are highly suggestive of ANE and help distinguish it from other causes of acute encephalopathy, including infectious, metabolic, and autoimmune etiologies.

## Case presentation

A six-year-old girl presented to the emergency department with a several-day history of fever, progressive reduction in oral intake, and declining level of consciousness. According to her caregivers, she had initially developed a febrile illness associated with lethargy and poor feeding, which subsequently progressed to altered mental status. Shortly before presentation, she experienced seizure activity, prompting urgent medical evaluation. There was no documented history of recent trauma, toxin exposure, or previous neurological disorders. Her past medical history was significant only for glucose-6-phosphate dehydrogenase deficiency, with no known history of chronic neurological disease or developmental abnormalities.

Upon arrival, the patient appeared acutely ill and encephalopathic. Initial clinical assessment demonstrated altered consciousness with progressive neurological deterioration, raising concern for CNS involvement in the setting of an acute infectious illness. Given the severity of her presentation, she was admitted for urgent evaluation and subsequently transferred to the pediatric intensive care unit (PICU) for close neurological and cardiorespiratory monitoring. During the early phase of hospitalization, her neurological status continued to worsen, accompanied by recurrent seizures and progressive impairment of protective airway reflexes. Owing to respiratory compromise and declining neurological function, endotracheal intubation and invasive mechanical ventilation were required.

Comprehensive laboratory investigations were performed as part of the diagnostic work-up. The results demonstrated evidence of systemic inflammation and associated metabolic derangements. Microbiological investigations were undertaken to identify an infectious etiology, and respiratory viral testing confirmed Influenza B infection. Given the acute onset of encephalopathy in association with a confirmed viral illness, an extensive evaluation was conducted to exclude alternative causes of acute neurological deterioration, including infectious encephalitis, metabolic encephalopathy, toxic exposure, autoimmune encephalitis, and other inflammatory disorders of the CNS.

Non-contrast CT of the brain demonstrated bilateral symmetrical hypodensities involving both external capsules (Figure 1). In view of the concerning neurological findings and ongoing clinical deterioration, an MRI of the brain was subsequently performed. MRI revealed extensive symmetrical hyperintense signal abnormalities on T2-weighted and fluid-attenuated inversion recovery sequences involving the periventricular white matter, bilateral thalami, external capsules, brainstem, and cerebellum. These lesions demonstrated associated diffusion restriction without evidence of post-contrast enhancement (Figure 2). The characteristic pattern of bilateral, symmetrical thalamic and multifocal white matter involvement, together with the clinical presentation following Influenza B infection, was highly suggestive of ANE. Following a multidisciplinary review of the clinical, laboratory, microbiological, and radiological findings, a diagnosis of Influenza B-associated ANE was established.

**Figure 1:**
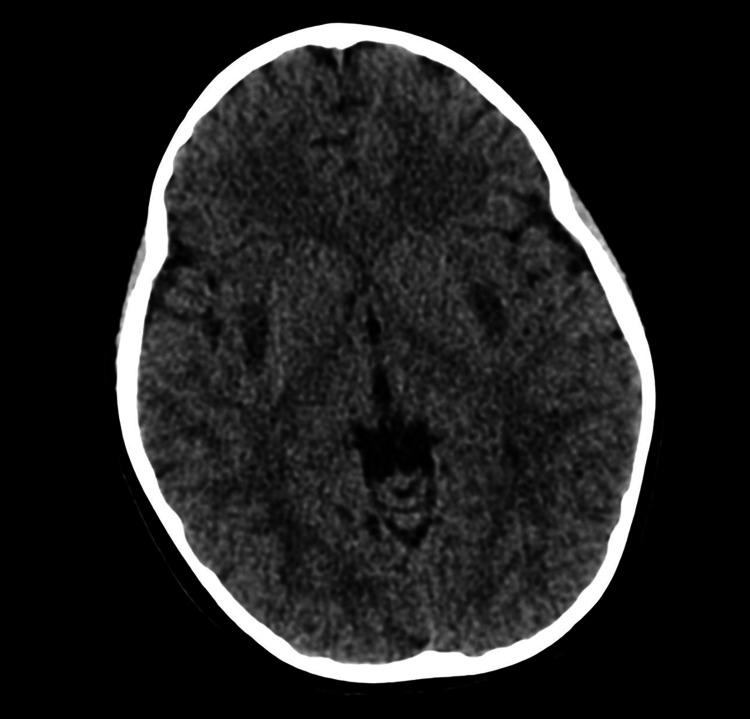
Axial CT brain showing bilateral hypodensities in the putamina. Axial non-contrast CT of the brain at presentation demonstrates bilateral symmetric hypodensities involving the putamina, suggestive of early imaging features seen in acute necrotizing encephalopathy in the appropriate clinical context.

**Figure 2:**
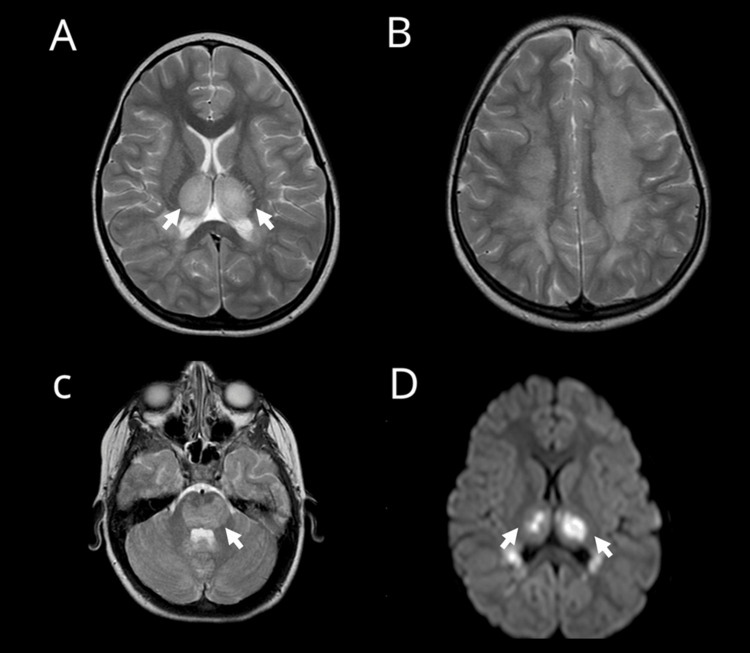
Axial MRI brain showing multifocal abnormalities involving the thalami, white matter, and brainstem with diffusion restriction. Axial MRI of the brain demonstrates characteristic findings of acute necrotizing encephalopathy. (A-C) Axial T2-weighted images show bilateral thalamic swelling with increased signal intensity (arrows) (A); confluent symmetric hyperintensities in the periventricular white matter of the centrum semiovale bilaterally (B); and increased signal intensity involving the central pons (arrow) (C). (D) Diffusion-weighted imaging (DWI) demonstrates restricted diffusion within the bilateral thalami (arrows), in keeping with cytotoxic edema and acute injury.

Given the life-threatening nature of the disease and the risk of permanent neurological injury, aggressive immunomodulatory treatment was initiated promptly. The patient received high-dose corticosteroid therapy in combination with intravenous immunoglobulin (IVIG). Empirical broad-spectrum antimicrobial therapy was also administered while infectious causes requiring specific treatment were being evaluated. Supportive management in the PICU included invasive mechanical ventilation, hemodynamic monitoring, fluid and electrolyte optimization, nutritional support, and comprehensive critical care measures. Seizure activity was managed with multiple antiseizure medications, resulting in gradual control of epileptic events. The patient also developed dystonic movements during the course of her illness, which necessitated additional pharmacological management and close neurological follow-up.

The patient’s PICU course was further complicated by several critical care-related conditions. She developed ventilator-associated pneumonia, which was treated according to microbiological findings and institutional antimicrobial protocols. In addition, intermittent cardiac arrhythmias were observed during her hospitalization and were closely monitored by the pediatric cardiology team. She also experienced transient electrolyte imbalances that required correction and serial biochemical monitoring. Despite these complications, her overall condition gradually improved with ongoing multidisciplinary management.

Over time, progressive neurological recovery was noted, accompanied by stabilization of respiratory function. This allowed successful weaning from mechanical ventilation and eventual extubation without major respiratory sequelae. Serial neurological assessments demonstrated gradual improvement in her level of consciousness and clinical status. Follow-up neuroimaging showed interval regression of the previously identified lesions, with evidence of residual chronic changes consistent with the evolution of ANE. Following stabilization and completion of the acute phase of treatment, the patient was transferred from the PICU to the general pediatric ward for continued multidisciplinary care, rehabilitation, and neurological follow-up. Ongoing rehabilitation efforts focused on optimizing neurological recovery, functional status, and long-term developmental outcomes.

## Discussion

ANE is a rare but severe form of acute encephalopathy with global distribution, typically preceded by a viral prodrome followed by rapid neurological deterioration. Most commonly associated with influenza and other respiratory viruses, its exact pathogenesis remains incompletely understood, although both host susceptibility and aberrant immune activation are believed to play central roles. The majority of cases are sporadic and non-recurrent; however, familial and recurrent forms have been described, highlighting important biological heterogeneity [4].

Genetic predisposition has been increasingly recognized, particularly mutations in the RANBP2 gene, which define familial ANE1 inherited in an autosomal dominant pattern with incomplete penetrance [5]. Additional susceptibility genes, including CPTII and SCN1A, as well as HLA associations such as HLA-DRB11401, HLA-DRB30202, and HLA-DQB1*0502, further support a role for host immune regulation in disease expression. Pathophysiologically, ANE is thought to result from an exaggerated cytokine-mediated immune response triggered by viral infection, leading to blood-brain barrier disruption and multifocal brain injury. Elevated levels of pro-inflammatory mediators, including interleukin-6 and tumor necrosis factor-α, have been demonstrated in serum and cerebrospinal fluid [6]. Clinically, neurological decline typically occurs within a short interval, often approximately three days after the onset of prodromal symptoms [6].

Neuroimaging is the cornerstone of diagnosis and prognostication. The characteristic finding is multifocal, symmetric lesions involving the bilateral thalami, brainstem, cerebellum, and cerebral white matter on CT and MRI [4]. Associated hemorrhage and extensive necrosis are generally linked to poorer outcomes. Although ANE predominantly affects children under five years of age, adult cases, though rare, are increasingly recognized. First described by Mizuguchi in 1995, ANE encompasses sporadic, recurrent, viral-associated, and genetically mediated forms, including those linked to SARS-CoV-2 infection. Early differentiation from mimics such as acute disseminated encephalomyelitis is critical, as management strategies and prognostic implications differ substantially.

Despite advances in critical care and immunomodulatory therapy, outcomes remain poor overall, with approximately two-thirds of patients experiencing death or significant neurological disability [7]. Early administration of high-dose corticosteroids, particularly within 24 hours of symptom onset, has been associated with improved outcomes in selected patients without brainstem involvement [1]. However, other therapeutic modalities, including intravenous immunoglobulin, plasmapheresis, and antiviral agents, have not demonstrated consistent efficacy [8]. Survivors often experience long-term neurological sequelae, and prognosis is particularly unfavorable in cases involving the brainstem or extensive hemorrhagic lesions. Preventive strategies, including annual influenza vaccination and early antiviral therapy during respiratory illness in at-risk individuals, remain important considerations for reducing disease burden [7].

## Conclusions

ANE is a rare but severe neurological complication most commonly associated with viral infections, particularly influenza. This case illustrates the fulminant course of Influenza B-associated ANE in a previously healthy child, resulting in severe encephalopathy, status epilepticus, and prolonged critical illness requiring intensive care support.

Early recognition of neurological deterioration, timely neuroimaging, and prompt multidisciplinary management were crucial in stabilizing the patient and mitigating further systemic and neurological complications. Despite aggressive supportive care and immunomodulatory therapy, the patient developed significant neurological sequelae, highlighting the devastating potential outcomes of this condition even with advanced critical care.

This case emphasizes the importance of maintaining a high index of suspicion for ANE in children presenting with encephalopathy following influenza infection. While early diagnosis and intervention may improve survival and functional outcomes, long-term neurological impairment remains common. Further research is required to better define optimal therapeutic strategies and prognostic indicators in influenza-associated ANE.
